# Murray law-based quantitative flow ratio to assess left main bifurcation stenosis: selecting the angiographic projection matters

**DOI:** 10.1007/s10554-023-02974-z

**Published:** 2023-10-23

**Authors:** Nozomi Kotoku, Kai Ninomiya, Daixin Ding, Neil O’Leary, Akihiro Tobe, Kotaro Miyashita, Shinichiro Masuda, Shigetaka Kageyama, Scot Garg, Jonathon A. Leipsic, Saima Mushtaq, Daniele Andreini, Kaoru Tanaka, Johan de Mey, William Wijns, Shengxian Tu, Nicolo Piazza, Yoshinobu Onuma, Patrick W. Serruys

**Affiliations:** 1 Department of Cardiology, University of Galway, University Road, Galway, H91 TK33 Ireland; 2 The Lambe Institute for Translational Medicine, The Smart Sensors Laboratory and CURAM, University of Galway, Galway, Ireland; 3 Department of Cardiology, Royal Blackburn Hospital, Blackburn, UK; 4 Department of Medicine and Radiology, University of British Columbia, Vancouver, BC Canada; 5 Departments of Cardiovascular Imaging and Surgery, Centro Cardiologico Monzino, IRCCS, Milan, Italy; 6 Division of Cardiology and Cardiac Imaging, IRCCS Galeazzi Sant’Ambrogio, Milan, Italy; 7 Department of Biomedical and Clinical Sciences, University of Milan, Milan, Italy; 8 Department of Radiology, Universitair Ziekenhuis Brussel, VUB, Brussels, Belgium; 9 School of Biomedical Engineering, Shanghai Jiao Tong University, Shanghai, China; 10 Department of Medicine, Division of Cardiology, McGill University Health Center, Montreal, QC Canada

**Keywords:** Bifurcation lesion, Computed tomography, Coronary angiography, Fractional flow reserve, Left main coronary artery disease, Murray law-based quantitative flow ratio

## Abstract

Murray law-based quantitative flow ratio (µQFR) assesses fractional flow reserve (FFR) in bifurcation lesions using a single angiographic view, enhancing the feasibility of analysis; however, accuracy may be compromised in suboptimal angiographic projections. FFR_CT_ is a well-validated non-invasive method measuring FFR from coronary computed tomographic angiography (CCTA). We evaluated the feasibility of µQFR in left main (LM) bifurcations, the impact of the optimal/suboptimal fluoroscopic view with respect to CCTA, and its diagnostic concordance with FFR_CT_. In 300 patients with three-vessel disease, the values of FFR_CT_ and µQFR were compared at distal LM, proximal left anterior descending artery (pLAD) and circumflex artery (pLCX). The optimal viewing angle of LM bifurcation was defined on CCTA by 3-dimensional coordinates and converted into a 2-dimensional fluoroscopic view. The best fluoroscopic projection was considered the closest angulation to the optimal viewing angle on CCTA. µQFR was successfully computed in 805 projections. In the best projections, µQFR sensitivity was 88.2% (95% CI 76.1–95.6) and 84.8% (71.1–93.7), and specificity was 96.8% (93.8–98.6) and 97.2% (94.4–98.9), in pLAD and pLCX, respectively, with regard to FFR_CT_. The AUC of µQFR for predicting FFR_CT_ ≤ 0.80 tended to be improved using the best versus suboptimal projections (0.94 vs. 0.89 [p = 0.048] in pLAD; 0.94 vs. 0.88 [p = 0.075] in pLCX). Computation of µQFR in LM bifurcations using a single angiographic view showed high feasibility from post-hoc analysis of coronary angiograms obtained for clinical purposes. The fluoroscopic viewing angle influences the diagnostic performance of physiological assessment using a single angiographic view.

**Supplementary Information:**

The online version contains supplementary material available at 10.1007/s10554-023-02974-z.

## Introduction

In patients with complex coronary artery disease (CAD), the presence or absence of left main (LM) disease (LMCAD) is an important prognostic factor in deciding between percutaneous coronary intervention (PCI) and coronary artery bypass grafting (CABG). Functional assessment of coronary stenoses has become the standard of care to evaluate the significance of coronary flow-limitation, and to justify PCI in contemporary practice [1]. Imaging-derived physiological assessment based on invasive coronary angiography (ICA) or coronary computed tomographic angiography (CCTA) is an alternative to wire-based pressure measurements, and offers the benefits of being less invasive, more cost-effective, and having a shorter procedure time. Fractional flow reserve (FFR) derived from CCTA (FFR_CT_) is a well-established non-invasive method based on three-dimensional (3D) finite element analysis, Navier–Stokes equation, and computational fluid dynamics [2].

The LM bifurcation encompasses the LM shaft, the proximal left anterior descending (LAD) artery, and the proximal left circumflex artery (LCX), creating a 3D structure that is rarely in one plane [3]. It follows that projecting the 3D LM bifurcation structure onto a 2D angiographic projection will inevitably cause foreshortening and overlap, and consequently evaluating it by quantitative coronary angiography (QCA) is frequently inaccurate.

Furthermore, the step-down phenomenon in diameters between LM and its daughter branches can lead to inappropriate calculation of reference diameters in the quantitative assessment of the bifurcation lesion [4, 5]. The Murray law-based quantitative flow reserve ratio (µQFR) is a novel computational method applied to a single ICA view that takes into account side branch diameters to compute fractal flow division [6].

The first validation study reported that computation of µQFR using an optimal projection had an area under the receiver operating characteristic curve (AUC) of 0.97 for predicting a pressure-derived FFR ≤ 0.80, but its diagnostic accuracy was reduced with sub-optimal projections (AUC 0.92, difference 0.05, p < 0.001) [6]. The method of selecting the optimal projection was not described in that seminal publication [6] and it remains unclear what the actual impact of the fluoroscopic viewing angle is on the µQFR, especially in complex anatomy such as the LM bifurcation.

The first objective of this study was to evaluate the feasibility of µQFR in assessing LM bifurcation lesions and its concordance with FFR_CT_ in patients with complex CAD. The second objective was to investigate the variation of µQFR values according to various selected angiographic views and the impact of selecting the optimal/suboptimal projection.

## Methods

### Study design

This study used the pooled paired dataset of ICA and CCTA from 303 patients with three-vessel disease (3VD) with or without LMCAD from the sub-study of SYNTAX (SYNergy between percutaneous coronary intervention with TAXus and cardiac surgery) II trial (n = 51), SYNTAX III REVOLUTION trial (n = 192), and FASTTRACK CABG trial (n = 60). The protocol design and results of each trial have been reported previously [7–11]. Baseline µQFR and FFR_CT_ were assessed, and the optimal viewing angle was defined by CCTA. CCTA image acquisition detail is in Supplementary Methods 1. The study protocol was approved at each enrolling site by the institutional review board or ethics committee.

**Fig. 1:**
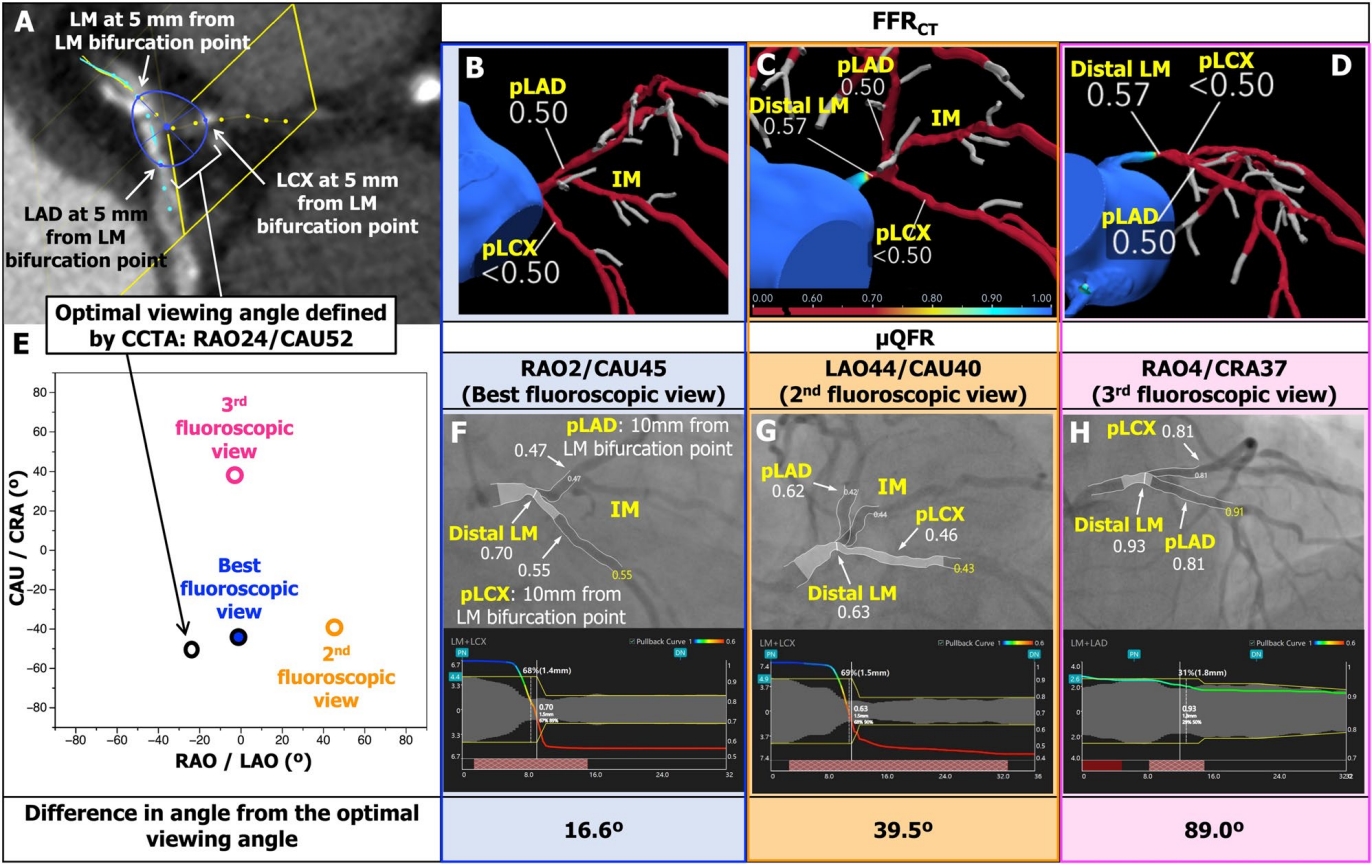
Example of image analyses of CCTA (**A**), FFR_CT_ (**B**–**D**), and µQFR (**E**–**H**). The optimal viewing angle of LM bifurcation was defined on CCTA analysis (**A**). The best fluoroscopic view was defined as the closest X-ray gantry angulation to the optimal angle defined by CCTA (**E**). Matched views of the FFR_CT_ and angiography by µQFR were presented in panels **B**–**D** and **F–H**. *CAU* caudal, *CCTA* coronary computed tomographic angiography, *CRA* cranial, *FFR*_*CT*_ fractional flow reserve derived from computed tomography, *LAO* left anterior oblique, *LM* left main coronary artery, *pLAD* proximal left anterior descending artery 10 mm distal to the LM bifurcation point, *pLCX* proximal left circumflex artery 10 mm distal to the LM bifurcation point, *RAO* right anterior oblique, *µQFR* Murray law-based quantitative flow reserve ratio

For physiological assessment of LM bifurcation by FFR_CT_ and µQFR, three fiducial anatomical landmark points were considered: (i) distal LM; (ii) proximal LAD 10 mm distal to the LM bifurcation point (pLAD); (iii) proximal LCX 10 mm distal to the LM bifurcation point (pLCX) (Fig. 1, Supplementary Fig. 1). Up to 3 single-fluoroscopic projections with adequate contrast filling but excluding projections with obvious overlap or foreshortening in LM, pLAD, and pLCX, were analysed with µQFR (Fig. 1, Supplementary Fig. 2). The “optimal viewing angle” of the LM bifurcation was defined on CCTA analysis, whilst the “best fluoroscopic view” was defined as the projection with closest X-ray gantry angulation to the “optimal viewing angle defined by CCTA.” Similarly, the projection with the second and third closest angulation to the “optimal viewing angle defined by CCTA” was defined as the “2nd- and 3rd fluoroscopic view”, respectively (Fig. 1).

### Analysis of CCTA to define the optimal viewing angle

To define the “optimal viewing angle”, CCTA was analysed using FluoroCT version 3.2 (Circle Cardiovascular Imaging, Calgary, Alberta, Canada). Centerlines were created from LM to LAD and LCX at least 5 mm proximally and distally from LM bifurcation point using curved multiplanar reconstruction (Supplementary Fig. 3). The optimal viewing angle of the LM bifurcation, which is perpendicular to the *en face* plane created by 3 dots in LM, LAD, and LCX at 5 mm from the LM bifurcation point, was calculated by the following formula, embedded in the FluoroCT application:

$$\mathrm{\varnothing }=-\mathrm{arctan}\left[\frac{\mathrm{cos}\left(\theta -\theta en face\right)}{\mathrm{tan\varnothing }en face}\right]$$

where $\mathrm{\varnothing }$ is the cranio-caudal (CRA/CAU) angle of the optimal viewing angle at right anterior oblique/left anterior oblique (RAO/LAO) angle $\theta$, and $\mathrm{\varnothing }en face$ and $\theta en face$ are, respectively, the CRA/CAU and RAO/LAO angles of the structure viewed *en face* [12, 13].

### Analysis of FFR_CT_

FFR_CT_ was performed by HeartFlow, Inc. (Redwood City, California), blinded to angiographic data. A quantitative 3D anatomic model of the aortic root and epicardial coronary arteries was generated from the CCTA images for each patient. Coronary blood flow and pressure were computed under conditions simulating maximal hyperemia [2, 14]. A cut-off FFR_CT_ ≤ 0.80 was used to indicate significant flow-limitation [15].

### Analysis of µQFR

In the independent core laboratory (CORRIB Core Lab, Galway, Ireland), µQFR analysis was performed using AngioPlus Core software (version V2, Pulse Medical, Shanghai, China) [6]. Methods to compute µQFR are described in Supplementary Methods 2. Contrast flow velocity was automatically converted to hyperemic flow velocity, and pressure drop was calculated using fluid dynamics equations (6). A cut-off µQFR ≤ 0.80 was used to indicate significant flow-limitation [6].

### Bifurcation QCA analysis

In the independent core laboratory (CORRIB Core Lab, Galway, Ireland), bifurcation QCA analysis was performed using CAAS software (version 8.2, Pie Medical Imaging, Maastricht, The Netherlands) blinded to the µQFR and FFR_CT_.

### Intra- and inter- observer analysis

To assess intra- and inter-observer variability in µQFR analysis, 30 patients were randomly analysed twice by the same analyst with an interval of > 4 weeks and by a second analyst, following the same methods, with both blinded from each other and the previous computational results.

### Functional MEDINA classification

Functional MEDINA classes were defined as follows: (i) for distal LM (1, 0, 0), FFR_CT_/µQFR ≤ 0.80; (ii) for proximal LAD (0,1,0), ΔFFR_CT_/ΔµQFR (gradient between distal LM and pLAD) ≥ 0.06 [16]; (iii) for proximal LCX (0, 0, 1), ΔFFR_CT_/ΔµQFR (gradient between distal LM and pLCX) ≥ 0.06, respectively.

### Statistical analysis

Continuous variables are presented as mean and standard deviation (SD) or as median and interquartile range (IQR) depending on their distribution and compared using the Student’s t-test. Categorical variables are described as percentages and compared using chi-square test or Fisher exact, as appropriate. The Spearman’s correlation (rs) and the Passing–Bablok regression analysis were used to quantify the correlation between µQFR and FFR_CT_ [17]. Agreement between µQFR and FFR_CT_ was assessed by the Bland–Altman method, with plots for visual assessment accompanied by estimates of bias and 95% limits of agreement. Since FFR_CT_ does not provide actual values if < 0.50, an FFR_CT_ value of 0.50 was imputed in lesions with FFR_CT_ < 0.50 [14]. Similarly, in the case of total or sub-total occlusion, the FFR_CT_/µQFR value of 0.50 was imputed because FFR_CT_/µQFR cannot be measured in a totally occluded artery [14, 18]. In that case, the diameter stenosis value of 100% was imputed for bifurcation QCA assessment. To assess agreement between µQFR and FFR_CT_ according to the functional MEDINA classification, the percentage of the total agreement is reported using Cohen’s kappa statistic. The diagnostic performance of µQFR was quantified with FFR_CT_ ≤ 0.80 as a standard reference. AUC by the receiver-operating characteristic (ROC) curve analysis by Delong method was performed to compare the accuracy of µQFR computed in the best projections and suboptimal projections in predicting FFR_CT_ ≤ 0.80 [19]. The intra-observer and inter-observer reproducibility of µQFR was evaluated using the intraclass correlation coefficient (ICC). A 2-sided p-value < 0.05 was considered statistically significant. All statistical analyses were performed using R version 4.1.3 (R Foundation for Statistical Computing, Vienna, Austria) and SPSS version 27.0 (IBM Inc, Armonk, NY, USA).

## Results

Among the 303 patients, three had separate ostia of LAD and LCX, and were therefore excluded due to the absence of a LM bifurcation, leaving 300 LM bifurcations in the study. Baseline patient characteristics are shown in Table 1. A total of 1621 angiographic projections were taken for the left coronary artery giving a mean number per patient of 5.4 (SD: 1.8) projections. Analysts aimed to analyse up to 3 projections for each LM bifurcation and deemed 805 (49.7%) of these projections to be of suitable quality (Supplementary Fig. 2 and Supplementary Fig. 4), and in all the µQFR of LM bifurcation was successfully computed.

**Table 1:** Baseline characteristics of study patients

Patient, % (number) or mean (standard deviation)	100 (300)
Male, % (n)	88.9 (265)
Age, year-old (SD)	66.8 (8.9)
Body mass index, kg/m^2^ (SD)	26.9 (4.3)
Current smoker, % (n)	20.1 (59)
Diabetes mellitus, % (n)	32.6 (97)
Insulin user, % (n)	7.7 (23)
Hypertension, % (n)	77.2 (230)
Dyslipidemia, % (n)	70.6 (207)
Previous stroke, % (n)	6.0 (18)
Previous myocardial infarction, % (n)	3.4 (10)
Family history of coronary artery disease, % (n)	33.1 (88)
COPD, % (n)	11.1 (33)
Peripheral vascular disease, % (n)	12.8 (38)
Left ventricular ejection fraction, % (SD)	55.2 (10.0)
Anatomical SYNTAX score derived from ICA (SD)	30.1 (11.2)
Anatomical SYNTAX score derived from CCTA (SD)	32.8 (12.1)

*CCTA* coronary computed tomographic angiography, *COPD* chronic obstructive pulmonary disease, *ICA* invasive coronary angiography

In patients who had ≥ 2 analysable projections, 17.7% (50/283) of patients had discordant of µQFR in different angiographic projections: one value being positive (≤ 0.80) and the other negative.

In the best projections, the median µQFR was 0.99 (IQR: 0.96–1.00; n = 300), 0.96 (0.85–0.98), and 0.95 (0.87–0.98) in distal LM, pLAD, and pLCX, respectively. The median FFR_CT_ was 0.97 (IQR: 0.94–0.99; n = 300), 0.93 (0.86–0.96), and 0.94 (0.87–0.97) in distal LM, pLAD, and pLCX, respectively. The distribution of µQFR and FFR_CT_ in each anatomical landmark point is illustrated as a histogram in Supplementary Fig. 5.

The distribution of functional MEDINA classes on FFR_CT_ and µQFR in the best fluoroscopic view is reported in Supplementary Table 1, with the agreement in 61.0% (Kappa = 0.42).

### Optimal viewing angle for LM assessment on CCTA

**Fig. 2:**
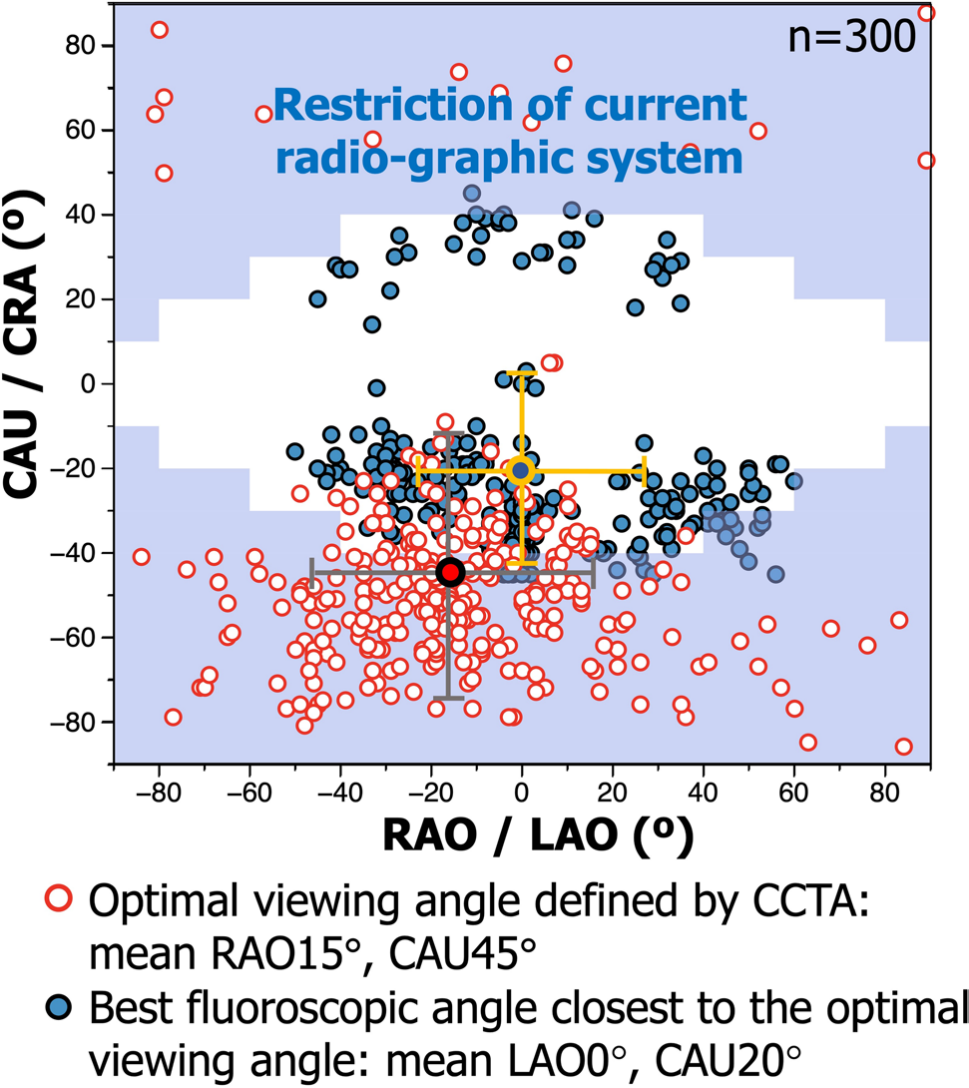
Optimal viewing angles and best fluoroscopic projections of 300 left main bifurcations. Red dots show optimal viewing angles defined by CCTA for each 300 LM bifurcation. Blue dots show the best fluoroscopic angles closest to the optimal viewing angle. Dots with cross show the mean angle (95%CI) respectively. According to the restriction of movement of current radiographic systems in the cath lab, a practical projection range was defined within limits described in **Supplementary Table 2** (12) (highlighted by stepped area). Abbreviations as in Fig. 1

On CCTA, the estimated optimal viewing angle for LM bifurcations was on average RAO15°, CAU45° (95% CI RAO44° to LAO15°, CAU16° to 75°, Fig. 2). On ICA, the best fluoroscopic viewing angle was on average LAO0°, CAU20° (95% CI RAO25° to LAO25°, CAU41° to CRA2°, Fig. 2). The mean difference between the optimal angle derived from CCTA and the best fluoroscopic angle selected from ICA was 30° (SD: 17): the 2nd fluoroscopic angle selected from ICA was 47° (SD: 19).

### Correlation and agreement between FFR_CT_ and µQFR on LM assessment

**Fig. 3:**
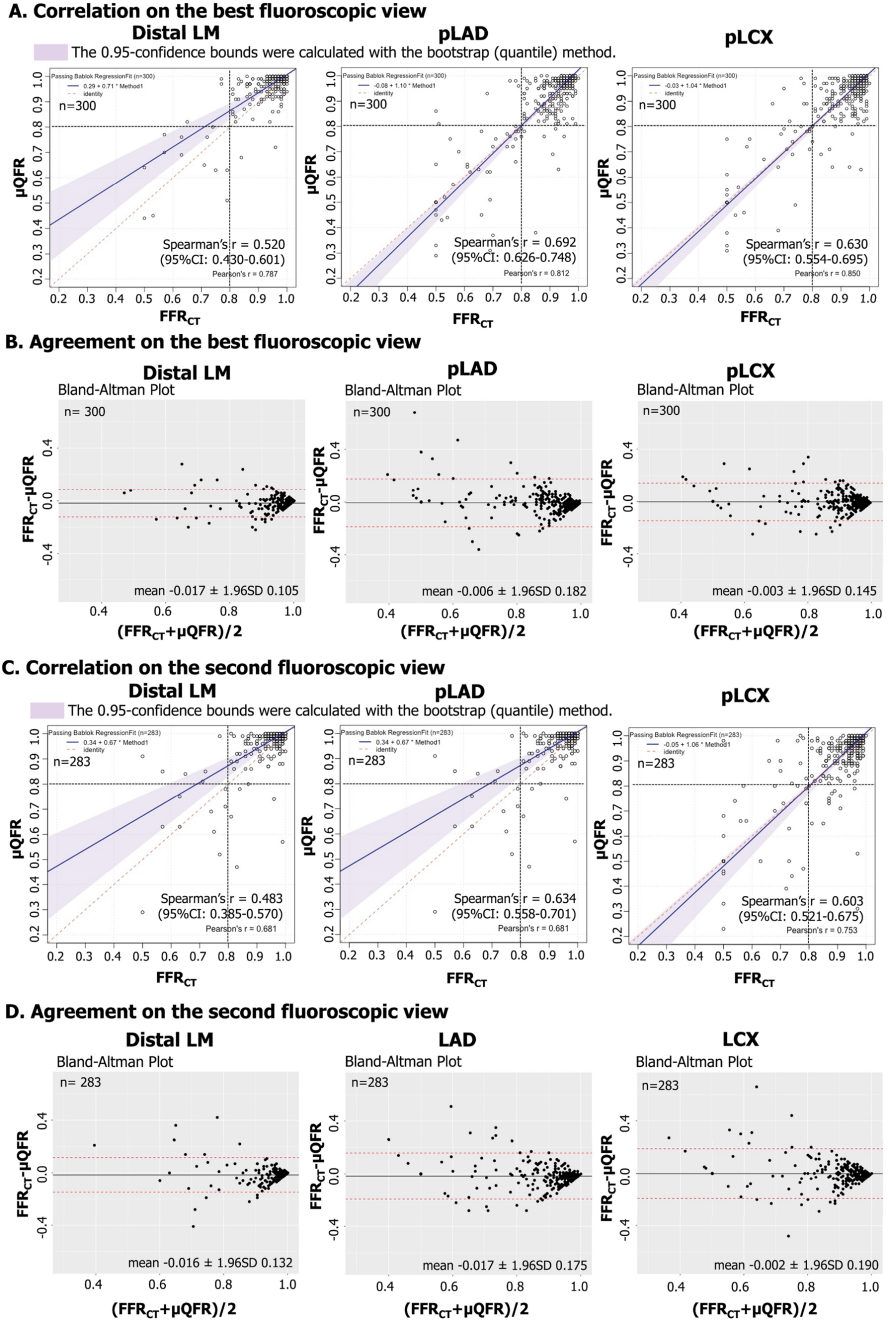
Correlation and agreement between FFR_CT_ and µQFR on LM bifurcation analysis on the best and 2nd fluoroscopic view. Abbreviations as in Fig. 1

The correlation and agreement between µQFR assessed in the best fluoroscopic view and FFR_CT_ for LM assessments are shown in Fig. 3A and B. In the best fluoroscopic view, Spearman’s correlation coefficient demonstrated a moderate correlation in distal LM (rs = 0.520, 95% CI 0.430–0.601), and a strong correlation in pLAD (rs = 0.692, 95% CI 0.626–0.748) and pLCX (rs = 0.630, 95% CI 0.554–0.695). The Bland–Altman analysis between µQFR and FFR_CT_ demonstrated slightly higher values with µQFR in all three measurement sites, with a mean difference in the best fluoroscopic view of − 0.017 (1.96SD: 0.105), − 0.006 (1.96SD: 0.182), and − 0.003 (1.96SD: 0.145), at distal LM, pLAD, and pLCX, respectively. Bland–Altman plots and limits calculated on a log scale are shown in Supplementary Fig. 6, considering that spread of the differences increases with decreasing mean of the observations [20].

### Diagnostic concordance between FFR_CT_ and µQFR in the best fluoroscopic view

The diagnostic concordance between FFR_CT_ and µQFR is summarized in Table 2; estimates of discrimination need to be interpreted with caution given the low number of cases of LM bifurcation disease with FFR_CT_ ≤ 0.80 (16 [5.3%], 52 [17.3%], and 46 [15.3%] in distal LM, pLAD, and pLCX, respectively). This limitation can be observed by particularly wide confidence intervals of the estimated sensitivity of µQFR.

**Table 2:** Diagnostic performance of µQFR on LM bifurcation assessment with FFR_CT_ ≤ 0.80 as a standard reference

	Distal LM	pLAD	pLCX
Best fluoroscopic view (n = 300)			
Accuracy	98.3% (96.2–99.5) (295/300)	95.3% (92.3–97.4) (286/300)	95.3% (92.3–97.4) (286/300)
Sensitivity	81.2% (54.4–96.0) (13/16)	88.2% (76.1–95.6) (45/51)	84.8% (71.1–93.7) (39/46)
Specificity	99.3% (97.5–99.9) (282/284)	96.8% (93.8–98.6) (241/249)	97.2% (94.4–98.9) (247/254)
PPV	86.7% (59.5–98.3) (13/15)	84.9% (72.4–93.3) (45/53)	84.8% (71.1–93.7) (39/46)
NPV	98.9% (97.0–99.8) (282/285)	97.6% (94.8–99.1) (241/247)	97.2% (94.4–98.9) (247/254)
+ LR	115.38 (28.43–468.31)	27.46 (13.79–54.70)	30.76 (14.67–64.53)
−LR	0.19 (0.07–0.52)	0.12 (0.06–0.26)	0.16 (0.08–0.31)
Apparent prevalence (µQFR)	5.0% (2.9–8.1)	17.7% (13.5–22.5)	15.3% (11.4–19.9)
True prevalence (FFR_CT_)	5.3% (3.1–8.5)	17.0% (12.9–21.7)	15.3% (11.4–19.9)
Bifurcation QCA^a^			
DS ≥ 50%, % (n)	5.3% (14)	9.1% (24)	9.9% (26)
MLA, mm (SD)	3.18 (0.88)	2.13 (0.82)	1.97 (0.73)
RVD, mm (SD)	4.06 (0.82)	2.78 (0.71)	2.63 (0.62)
2nd fluoroscopic view (n = 283)			
Accuracy	95.8% (92.7–97.8) (271/283)	90.1% (86.0–93.3) (255/283)	91.9% (88.1–94.8) (260/283)
Sensitivity	60.0% (32.3–83.7) (9/15)	69.6% (54.2–82.3) (32/46)	74.4% (58.8–86.5) (32/43)
Specificity	97.8% (95.2–99.2) (262/268)	94.1% (90.3–96.7) (223/237)	95.0% (91.4–97.4) (228/240)
PPV	60.0% (32.3–83.7) (9/15)	69.6% (54.2–82.3) (32/46)	72.7% (57.2–85.0) (32/44)
NPV	97.9% (95.2–99.2) (262/268)	94.1% (90.3–96.7) (223/237)	95.4% (91.9–97.7) (283/239)
+ LR	27.80 (10.98–65.43)	11.78 (6.84–20.27)	14.88 (8.35–26.55)
−LR	0.41 (0.22–0.76)	0.32 (0.21–0.50)	0.27 (0.16–0.45)
Apparent prevalence (µQFR)	5.3% (3.0–8.6)	16.3% (12.2–21.1)	15.5% (11.5–20.3)
Bifurcation QCA^a^			
DS ≥ 50%, % (n)	5.9% (14)	9.2% (22)	10.1% (24)
MLA, mm (SD)	3.12 (0.86)	2.04 (0.74)	1.98 (0.73)
RVD, mm (SD)	4.00 (0.81)	2.71 (0.61)	2.63 (0.61)

Values are proportions in % (95% confidence interval)

*DS* diameter stenosis *LM* left main coronary artery, *MLD* minimal lumen diameter, NPV negative predicted value, *pLAD* proximal left anterior descending artery 10 mm distal to the LM bifurcation point, *pLCX* proximal left circumflex artery 10 mm distal to the LM bifurcation point, *PPV* positive predicted value, *QCA* quantitative coronary angiography, *RVD* reference vessel diameter, *µQFR* Murray law-based quantitative flow reserve, −LR negative likelihood ratio, *+LR* positive likelihood ratio

^a^Bifurcation QCA in LM, pLAD, and pLCX was analysable in 262, 264, and 263 vessels in the best fluoroscopic view, and 238, 240, and 238 vessels in the 2nd fluoroscopic view, respectively

**Fig. 4:**
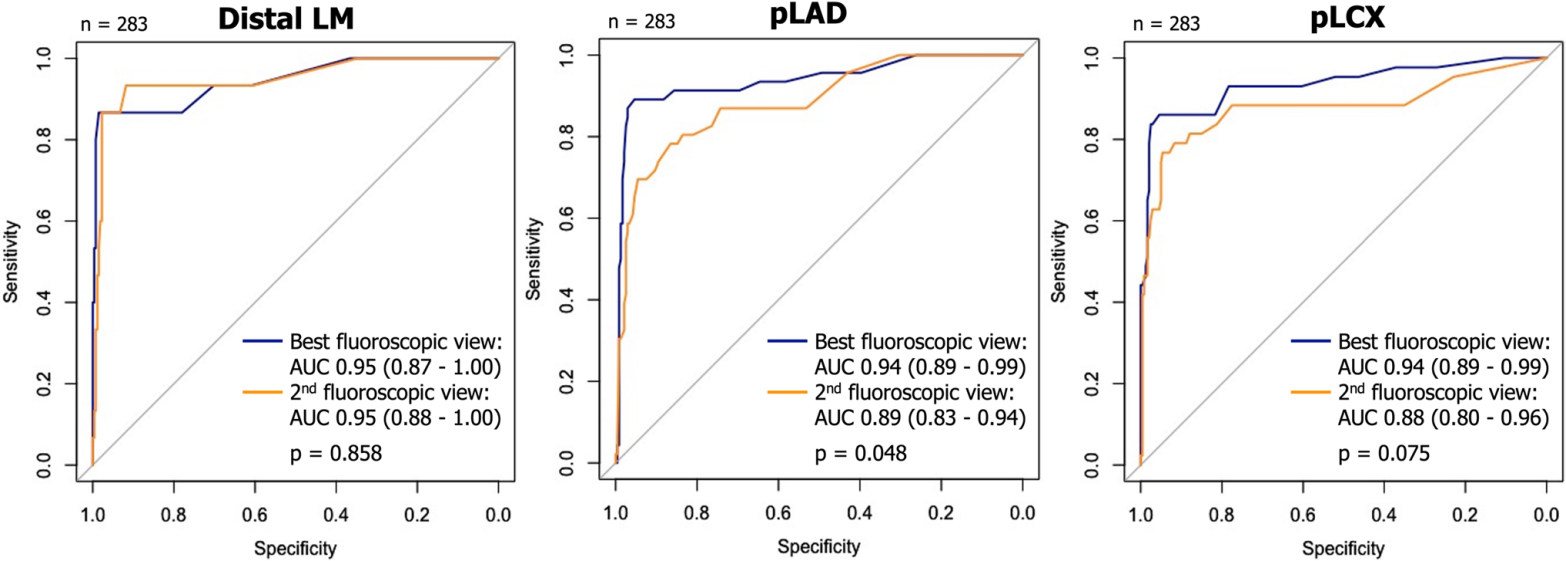
Comparison of ROC curves of µQFR between the best and 2nd fluoroscopic view with FFR_CT_ as a standard reference. The accuracy of µQFR in distal LM, pLAD, and pLCX was shown as the area under the curve (AUC) by the receiver-operating characteristic (ROC) curve of the best and 2nd fluoroscopic view in predicting FFR_CT_ ≤ 0.80, with the comparison between the best and 2nd fluoroscopic view by Delong method

In the best fluoroscopic view, diagnostic accuracy of µQFR was 98.3% (95% CI 96.2–99.5), 95.3% (95% CI 92.3–97.4), and 95.3% (95% CI 92.3–97.4), in distal LM, pLAD, and pLCX, respectively. Sensitivity in the best projections was 81.2% (95% CI 54.4–96.0), 88.2% (95% CI 76.1–95.6), and 84.8% (95% CI 71.1–93.7) in distal LM, pLAD, and pLCX, respectively. In the best projections, the AUC of µQFR for predicting an FFR_CT_ ≤ 0.80 was 0.95 (95% CI 0.87–1.00), 0.94 (95% CI 0.89–0.99), and 0.94 (95% CI 0.89–0.99), in distal LM, pLAD, and pLCX, respectively (Fig. 4).

### Correlation, agreement, and diagnostic concordance between FFR_CT_ and µQFR analysis in 2nd fluoroscopic views

The correlation and agreement between µQFR assessed in the 2nd fluoroscopic view and FFR_CT_ for LM assessments are shown in Fig. 3C and D (Supplementary Fig. 6B) and Supplementary Results 1.

Compared to the best fluoroscopic view, in the 2nd fluoroscopic view, the sensitivity of µQFR was relatively low at 60.0% (95% CI 32.3–83.7), 69.6% (95% CI 54.2–82.3), and 74.4% (95% CI 58.8–86.5) in distal LM, pLAD, and pLCX, respectively (Table 2). In the 2nd view, the AUC of µQFR for predicting FFR_CT_ ≤ 0.80 was 0.95 (95% CI 0.88–1.00, p = 0.858 compared to the best fluoroscopic view by Delong) in LM, 0.89 (95% CI 0.83–0.94, p = 0.048) in pLAD, and 0.88 (95% CI 0.80–0.96, p = 0.075) in pLCX, showing lower values in pLAD and pLCX than those in the best view (Fig. 4).

### Reproducibility of µQFR analysis on LM bifurcation assessment

Repeated µQFR analysis was performed on 30 patients, extracting values for 3 fiducial points of distal LM, pLAD, and pLCX (Supplementary Fig. 7). The ICC for intra-observer of µQFR was 0.91 (95% CI 0.81–0.96), 0.93 (95% CI 0.86–0.97), and 0.85 (95% CI 0.69–0.93), in distal LM, pLAD, and pLCX, respectively. The ICC for inter-observer of µQFR was 0.95 (95% CI 0.90–0.98), 0.94 (95% CI 0.87–0.97), and 0.87 (95% CI 0.72–0.94) in distal LM, pLAD, and pLCX, respectively.

## Discussion

The main findings of the present study can be summarised as follows:

(1) µQFR of LM bifurcations derived from a single angiographic view was successfully computed in all 805 analysed projections; (2) the selection of an appropriate/inappropriate fluoroscopic view reclassified the functional significance of µQFR (≤ 0.80 or > 0.80) in 17.7% of patients; (3) the AUC of µQFR for predicting an FFR_CT_ ≤ 0.80 tended to be better using the best versus 2nd fluoroscopic view (0.94 vs. 0.89 [p = 0.048] in pLAD; 0.94 vs. 0.88 [p = 0.075] in pLCX).

To overcome deficiencies of 2D QCA caused by converting a 3D structure into a 2D angiographic projection, 3D QCA was developed and used primarily in clinical research. In the RESEARCH and T-SEARCH registries, 3D QCA (CardiOp-B system version 2.1.0.151, Paieon Medical) of LM bifurcation lesions could only be analysed in 50.7% of patients due to the unavailability of two angiographic projections [21] (Supplementary Table 3). Similarly, in the TRYTON LM multi-centre registry, only 26.9% of paired pre- and post-PCI 3D QCAs (CAAS version 5.10, Pie Medical Imaging) of LM bifurcation lesions could be analysed [22], whilst in a sub-study of the SYNTAX trial, 75.1% of cases could be analysed (CardiOp-B system version 2.1.0.151, Paieon Medical) with as main reasons for non-feasible analysis overlap and/or tortuosity of branch vessels [23]. In Tomaniak et al.’s study on physiological assessment of LMCAD using 3D QCA-based vessel FFR (vFFR, CAAS8.1, Pie Medical Imaging), the main reason (60.7%) for screening failure was the insufficient quality of the ICA including substantial foreshortening of at least one of the two required optimal “most significant” views [24]. The computation of µQFR does not require a second projection, and therefore the likelihood of successful analysis is higher than with conventional angiography-derived FFR requiring two projections for 3D reconstruction.

The strong correlation of µQFR with FFR_CT_ was observed in both pLAD (rs = 0.692) and pLCX (rs = 0.630, Fig. 3A). In the best fluoroscopic view, diagnostic accuracy of µQFR for predicting FFR_CT_ ≤ 0.80 was excellent with AUC of 0.94 (95% CI 0.89–0.99) at both pLAD and pLCX (Fig. 4). The patient population was predominantly male (88.9%) in this study. Recently, it was reported that µQFR had comparable diagnostic performance between the sexes and significantly improved the detection of physiological significance, as defined by FFR, over angiography alone [25].

**Fig. 5:**
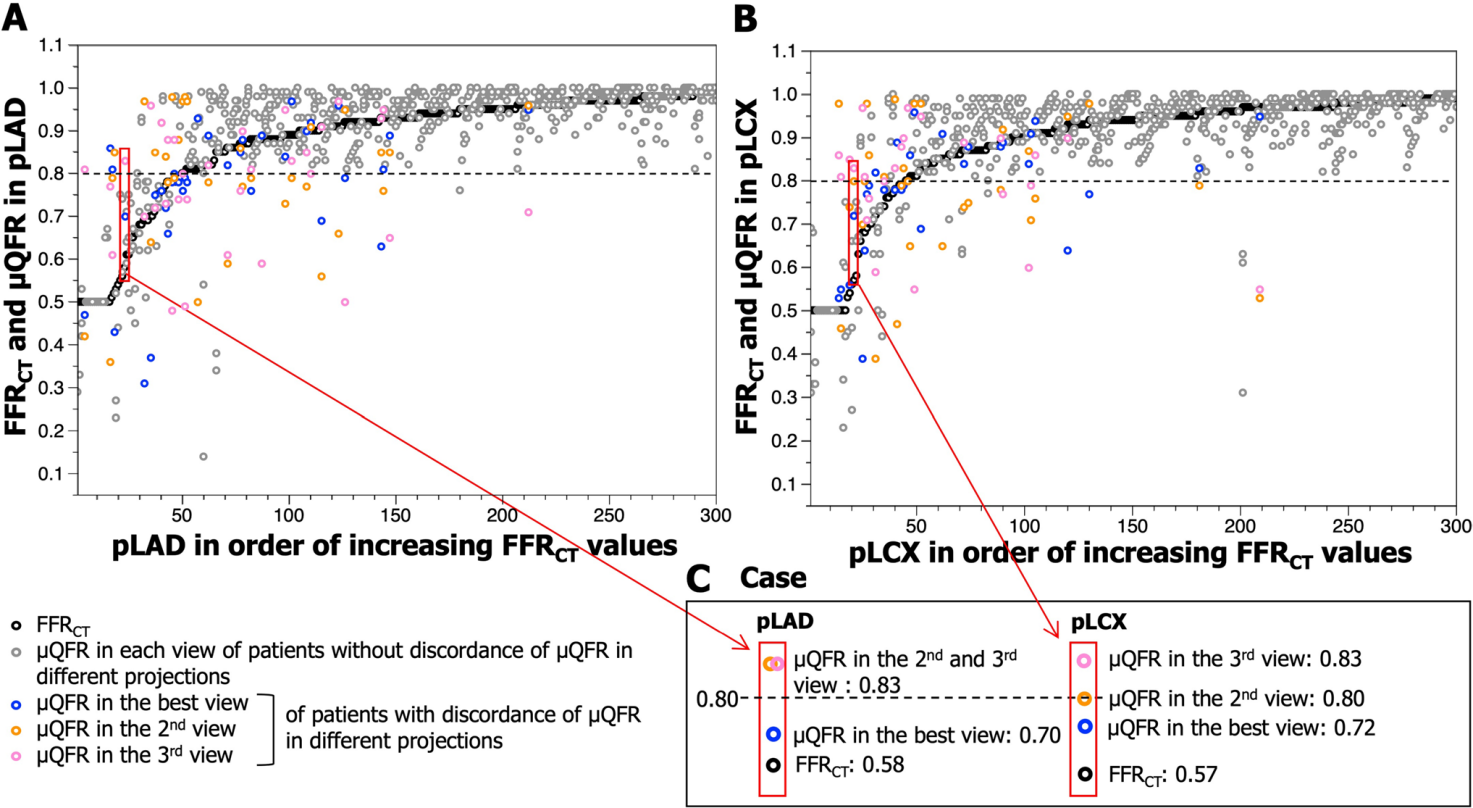
Variation of µQFR values in pLAD and pLCX in 805 projections of 300 patients. See description in “Discussion”. Abbreviations as in Fig. 1.

As shown in Fig. 5, the discrimination of functional significance of µQFR (≤ 0.80 or > 0.80) changed according to the selected angiographic projection. In the x-axis, 300 patients were sorted in ascending order of FFR_CT_ value of pLAD (Fig. 5A) and pLCX (Fig. 5B), respectively. FFR_CT_ and µQFR values in best and suboptimal projections for individual patients were plotted on the y-axis. µQFR values of the patient with discordance of µQFR in different angiographic projections—one value being positive (≤ 0.80) and the other negative—were displayed in color classified by the projections. On the other hand, both the best and suboptimal projections of cases without discordance of µQFR in different angiographic projections were displayed in gray. A case highlighted in the red frame represents the case where the significance of µQFR is influenced by the selection of the projection (Fig. 5C). In the best projection, µQFR was positive (≤ 0.80), which was consistent with the result of FFR_CT_. However, if the suboptimal projection was selected, µQFR value became falsely negative.

Whilst the use of a single angiographic view increases the feasibility of computing µQFR, its accuracy depends on the selection of the optimal angiographic projection. Patient-specific optimal fluoroscopic view for fluoroscopy-based FFR assessment could be determined from anatomic evaluation of CCTA prior to the fluoroscopic interventional procedure.

In the previous report, Kočka et al. analysed the LM bifurcation of 95 patients using CCTA and found that the mean optimal viewing angle for LM bifurcation was LAO 0°, CAU49° (95% CI: RAO 8° to LAO 8°, CAU 43° to 54°) [12]. In our study, the optimal viewing angle for LM bifurcation was on average RAO15°, CAU45° (95% CI RAO44° to LAO15°, CAU16° to 75°). The distribution of the optimal viewing angle for LM bifurcation (Fig. 2) was similar to those Kočka et al. reported with a widespread range of the RAO/LAO angle. Notably, only 20% (61/300) of patients was the optimal viewing angle obtainable in fluoroscopy due to the excessive caudal (or cranial) angulation of the X-ray gantry with the current hardware [12] (highlighted in Fig. 2 by stepped area), accompanied the considerable mean difference of 30 ± 17° between the optimal angle derived from CCTA and the best fluoroscopic angle selected from ICA. Notwithstanding this, the “best fluoroscopic view,” which was derived from “real-world” fluoroscopic projections retrospectively, tended to improve the AUC of µQFR analysis of LM bifurcations.

In previous literature, both necropsy studies and intracoronary imaging demonstrated that coronary lesions were often complex with markedly distorted or eccentric luminal shapes [26]. For a complicated coronary lesion such as LMCAD, any arbitrary angle of view could significantly misrepresent the extent of narrowing [26]. Considering the relatively low agreement (61%, Kappa = 0.42) of functional MEDINA classes on FFR_CT_ and µQFR, the best single view might be sufficient for a working projection, but not for diagnosis, especially for eccentric stenosis.

According to the recommendation of current guidelines, patients who have CCTA before going to the cath lab are increasing. In the future, the use of FFR_CT_ in clinical practice will also increase due to the latest evidence from FISH&CHIPS (FFRCT In Stable Heart disease & CTA Helps Improve Patient care and Societal costs) study, presented at the ESC congress 2023, which suggests that implementation of the FFR_CT_ program to a national level was associated with reduced mortality. In those cases, the pre-procedure physiological assessment would be done by FFR_CT_. Prior to the PCI procedure, CCTA as a “treatment planner” may facilitate the search for the most favourable fluoroscopic view that optimally exposes the bifurcation lesion to be treated, which will in turn reduce the number of exploratory injections of contrast medium and the amount of radiation needed to establish the “working projection,” for the procedure. Furthermore, post-PCI µQFR could be assessed in the optimal view to optimize the hemodynamic outcome post-procedure.

## Limitations

The present study must be interpreted with caution due to some limitations. First, invasive FFR as the gold standard of physiological assessment for intermediate coronary stenosis was not performed. A strong correlation between invasive FFR and FFR_CT_ has been previously reported in prospective trials [27–30], whereas greater AUC for QFR (QAngio XA 3D version 1.0.28.4, Medis Medical Imaging System) than that for FFR_CT_ has been also reported [31]. For LMCAD, there is no firm evidence to support the use of QFR (Medis Medical Imaging System), and in fact, the manufacturer does not recommend the QFR analysis on LM [32]. Therefore, we investigated the impact of optimal fluoroscopic angle on the correlation between µQFR—2D imaging physiological assessment and FFR_CT_—3D imaging physiological assessment in one of the most challenging lesion geometry, LM bifurcation.

Similarly, the cut-off value of FFR_CT_ and µQFR to identify hemodynamically significant coronary stenoses in the LM lesion has not been firmly established, although we used the classic cut-off value of ≤ 0.80. Patients with unprotected LMCAD treated medically have a 3-year mortality rate of 50% [33]. Additional physiological assessments of LMCAD beyond just the severity of stenosis, including µQFR and FFR_CT_ as well as invasive measures of FFR should provide additive prognostic information [34].

Second, this study was retrospective. The “best projection” was defined as the projection closest to the “optimal viewing angle” derived from CCTA, and analysed retrospectively. The impact of the optimal viewing angle predefined by CCTA for individual patients needs to be evaluated in a prospective study.

Third, accuracy needs to be cautiously interpreted since our sample size is limited to 300 patients, in particular the low number of cases with LMCAD. However, the prevalence of disease with an FFR_CT_ ≤ 0.80 in LM, pLAD, and pLCX is in keeping with the published literature [33]. Our population reflects the “real-world” or even a cohort of patients with more complex CAD anatomy; nevertheless, in the evaluation of the diagnostic performance of µQFR in LM bifurcation lesions, large-scale, prospective trials are warranted.

## Conclusions

The computation of µQFR in LM bifurcation analysis using a single angiographic view is highly feasible. A tailored optimal fluoroscopic view is essential for the physiological assessment of the LM bifurcation using a single angiographic view. CCTA planned prior to PCI may identify the best fluoroscopic view that will optimize exposure of the 3D bifurcation structure onto a 2D angiographic projection during the procedure.

### Supplementary Information

Below is the link to the electronic supplementary material.

Supplementary file1 (DOCX 1833 KB)

## Abbreviations

CAD: Coronary artery disease
CAU: Caudal
CCTA: Coronary computed tomographic angiography
CRA: Cranial
FFR: Fractional flow reserve
FFR_CT_: Fractional flow reserve derived from coronary computed tomographic angiography
LAD: Left anterior descending
LAO: Left anterior oblique
LCX: Left circumflex artery
LM: Left main coronary artery
PCI: Percutaneous coronary intervention
QCA: Quantitative coronary angiography
RAO: Right anterior oblique
µQFR: Murray law-based quantitative flow ratio
