# Supplementary material for: Murray law-based quantitative flow ratio to assess left main bifurcation stenosis: selecting the angiographic projection matters
Source: Int J Cardiovasc Imaging. 2023 Oct 23;40(1):195–206. doi: 10.1007/s10554-023-02974-z (PMC10774209; doi:10.1007/s10554-023-02974-z)

**Murray Law-based Quantitative Flow Ratio to Assess Left Main Bifurcation Stenosis: Selecting the Angiographic Projection Matters**

**Supplementary material**

**Supplementary Methods 1. CCTA acquisition**

CCTA was performed using scanners with at least 64-row detectors (256-row Somatom Definition Flash, Siemens, Munich, Germany; 320-row Aquilion One, Canon Medical Systems, Otawara, Japan; 64-row Brilliance, Philips, the Netherlands; 64-row Light Speed VCT and 256-slice Revolution CT, GE Healthcare, Milwaukee, WI, USA).

Nitrates was given prior to image acquisition and beta-blockers was used in patients with heart rate >65 beats/min. Tube settings depended on the patient body mass index (80 to 140 kV), and prospective ECG triggering was used for patients with lower heart rates to reduce radiation doses. Images were reconstructed using thin slices (0.50 to 0.67 mm) and medium smooth reconstruction filters in different phases.

**Supplementary Methods 2. Computation methods of µQFR**

The Murray law-based quantitative flow ratio (µQFR) was computed using the following methods:

(I) for the purposes of analysis, the analyst designated either left anterior descending (LAD) or left circumflex artery (LCX) to be the main analysis vessel. If a single stenosis was present in either vessel ≤10 mm from the bifurcation point, then that vessel was considered the main analysis vessel, and the other, the side branch. If both or neither vessel had a proximal stenosis, then the vessel with the least foreshortening by visual assessment was designated the main vessel.

(II) In the main analysis vessel, the analyst indicated the starting and stopping points for analysis. The main analysis vessel was analysed from the ostium of LM to an anatomical landmark (i.e., side branch) in a non-stenosed segment, to derive an accurate reference vessel diameter.

(III) The software automatically delineated the lumen contour of the main analysis vessel and all its side branches with diameters of ≥1.0 mm. The lumen contour was corrected manually if needed.

(IV) Contrast flow velocity was automatically converted to hyperemic flow velocity, and pressure drop was calculated using fluid dynamics equations (6). A cut-off µQFR ≤0.80 was used to indicate significant flow-limitation (6).

**Supplementary Results 1. Correlation and agreement between µQFR and FFR_CT_ in the 2^nd^ fluoroscopic views**

In the second view, Spearman’s correlation coefficient demonstrated a moderate correlation in distal LM (rs=0.483, 95%CI: 0.385-0.570), and a strong correlation in pLAD (rs=0.634, 95%CI: 0.558-0.701) and pLCX (rs=0.603, 95%CI: 0.521-0.675), showing lower estimated correlation values compared to those of the best fluoroscopic view. The Bland-Altman analysis between µQFR and FFR_CT_ demonstrated slightly higher values with µQFR in all three measurement sites, with a mean difference in the 2nd fluoroscopic view of -0.016 (1.96SD: 0.132), -0.017 (1.96SD: 0.175), and -0.002 (1.96SD: 0.190), at distal LM, pLAD, and pLCX, respectively.

**Supplementary Table 1. The distribution of functional MEDINA classes on FFR_CT_ and µQFR in the best fluoroscopic view**

Functional MEDINA classes were defined as follows: i) for the distal LM (1,0,0), FFR_CT_/µQFR ≤0.80; ii) for the proximal LAD (0,1,0), ΔFFR_CT_/ΔµQFR (gradient between the distal LM and pLAD) ≥0.06; iii) for the proximal LCX (0,0,1), ΔFFR_CT_/ΔµQFR (gradient between the distal LM and pLCX) ≥0.06, respectively. FFR_CT_ = Fractional flow reserve derived from computed tomography; LM = left main coronary artery; pLAD = proximal left anterior descending artery 10 mm distal to the LM bifurcation point; pLCX = proximal left circumflex artery 10 mm distal to the LM bifurcation point; µQFR = Murray law-based quantitative flow ratio.

| **FFR_CT_ Medina** | | | | | | | | | | |
| --- | --- | --- | --- | --- | --- | --- | --- | --- | --- | --- |
| **µQFR Medina** |  | **1,1,1** | **1,1,0** | **1,0,1** | **1,0,0** | **0,1,1** | **0,1,0** | **0,0,1** | **0,0,0** | **Total** |
|  | **1,1,1** | **3** | **1** | **0** | **1** | **2** | **0** | **0** | **0** | **7** |
|  | **1,1,0** | **0** | **1** | **0** | **1** | **0** | **0** | **0** | **0** | **2** |
|  | **1,0,1** | **0** | **0** | **0** | **2** | **0** | **0** | **0** | **0** | **2** |
|  | **1,0,0** | **0** | **0** | **0** | **4** | **0** | **0** | **0** | **0** | **4** |
|  | **0,1,1** | **1** | **0** | **0** | **0** | **11** | **8** | **8** | **8** | **36** |
|  | **0,1,0** | **0** | **0** | **0** | **0** | **6** | **23** | **4** | **13** | **46** |
|  | **0,0,1** | **0** | **0** | **0** | **1** | **5** | **7** | **21** | **22** | **55** |
|  | **0,0,0** | **0** | **0** | **0** | **1** | **3** | **17** | **6** | **120** | **148** |
|  | **Total** | **4** | **2** | **0** | **10** | **27** | **56** | **38** | **163** | **300** |
|  | **Cohen’s kappa = 0.42**  **Agreement = 61.0%** | | | | | | | | | |

**Supplementary Table 2. Definition of limitations of a practical projection range**

| **LAO or RAO angle** | **Maximum angle of CRA or CAU** |
| --- | --- |
| 0° to 40° | 40° |
| 41° to 60° | 30° |
| 61° to 80° | 20° |
| 81° to 90° | 10° |

CAU = caudal; CRA = cranial; LAO = left anterior oblique; RAO = right anterior oblique.

**Supplementary Table 3. Analysability of 3D QCA and 3D QCA-based FFR in previous studies for LMCAD**

| Trial | Analysis | Software | Analysability | Main reason for non-feasible analysis |
| --- | --- | --- | --- | --- |
| 3D QCA |  |  |  |  |
| RESEARCH and T-SEARCH registries^15^ | 3D QCA of LM bifurcation lesion | CardiOp-B system version 2.1.0.151, Paieon Medical | 50.7% | Unavailability of 2 angiographic projections |
| TRYTON LM multi-centre registry^16^ | Paired pre- and post-PCI 3D QCA | CAAS version 5.10, Pie Medical Imaging | 26.9% | Overlap and/or tortuosity of branch vessels |
| SYNTAX trial^17^ | Paired pre- and post-PCI 3D QCA | CardiOp-B system version 2.1.0.151, Paieon Medical | 75.1% | Overlap and/or tortuosity of branch vessels |
| 3D QCA-based FFR |  |  |  |  |
| Tomaniak et al.^18^ | 3D QCA-based vessel FFR of LMCA | vFFR, CAAS8.1, Pie Medical Imaging | 42.9% | Insufficient quality of the ICA including substantial foreshortening of at least one of the two required optimal views |

FFR = Fractional flow reserve; LMCAD = left main coronary artery disease; QCA = quantitative coronary angiography; 3D = three-dimensional.

**Supplementary Fig. 1 Definition of the left main bifurcation point in µQFR analysis**

The area surrounded by a green line shows a downsizing of the reference diameters between the LM and its daughter branch across the LM bifurcation according to Murray fractal law and was defined as the bifurcation core. Its proximal edge was defined as the distal LM and the LM bifurcation point. LM = left main coronary artery; pLAD = proximal left anterior descending artery 10 mm distal to the LM bifurcation point; pLCX = proximal left circumflex artery 10 mm distal to the LM bifurcation point; µQFR = Murray law-based quantitative flow reserve ratio.


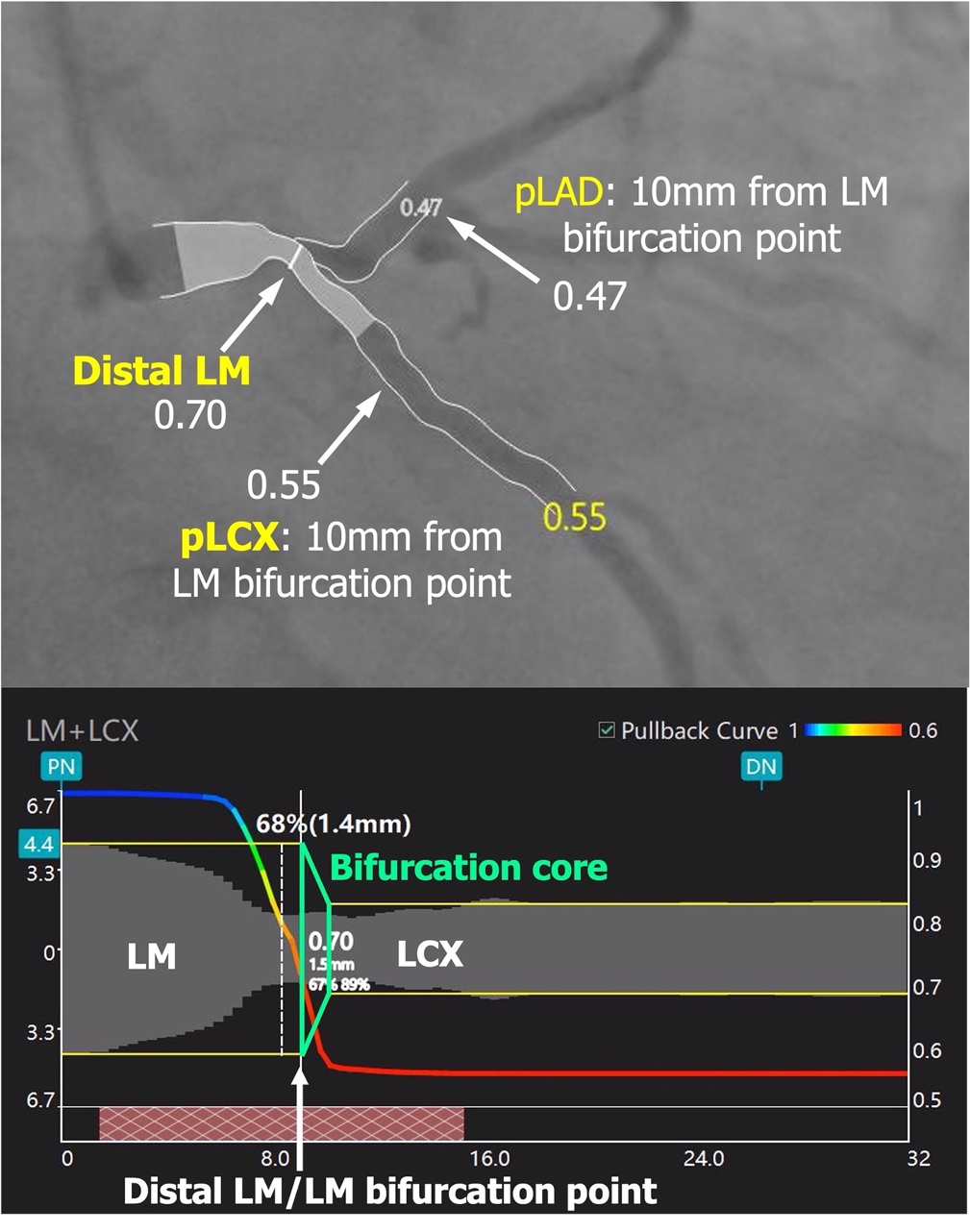


**Supplementary Fig. 2 Example of projections not selected for µQFR analysis of LM bifurcation**

Up to 3 single-projections with adequate contrast filling but excluding projections with obvious overlap of proximal LAD and LCX, or obvious foreshortening in LM, proximal LAD and, proximal LCX, were selected for analysis. Panel A and B show projections that were not selected during µQFR analysis in the case shown in **Fig. 1** due to **(A)** foreshortening in proximal LAD and overlap of proximal LAD and LCX, and **(B)** foreshortening in proximal LCX and overlap of distal LM and proximal LCX. CRA=cranial; LAD = left anterior descending artery; LAO = left anterior oblique; LCX = left circumflex artery; LM = left main coronary artery; RAO = right anterior oblique; µQFR = Murray law-based quantitative flow reserve ratio.


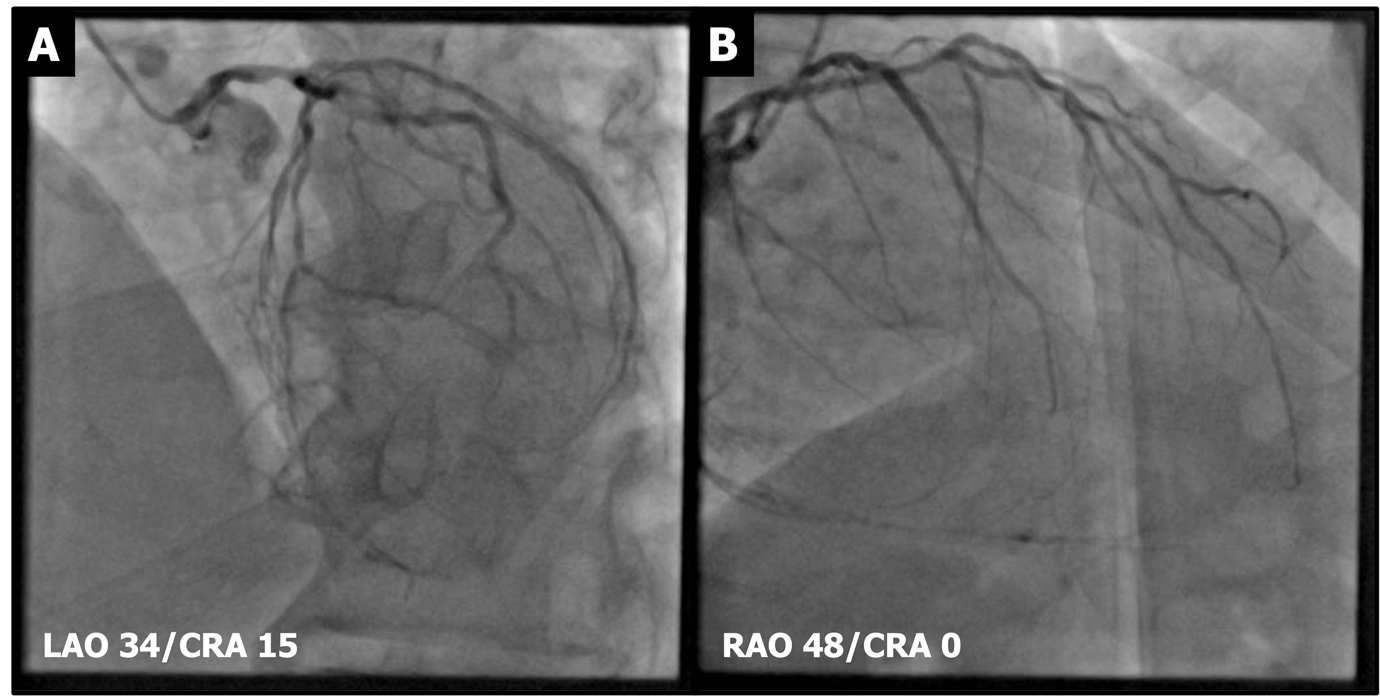


**Supplementary Fig. 3 Analysis of optimal fluoroscopic viewing angle for LM bifurcation derived from CCTA**

CAU = caudal; CCTA = coronary computed tomographic angiography; Other abbreviations as in **Supplementary Fig. 2**.

**
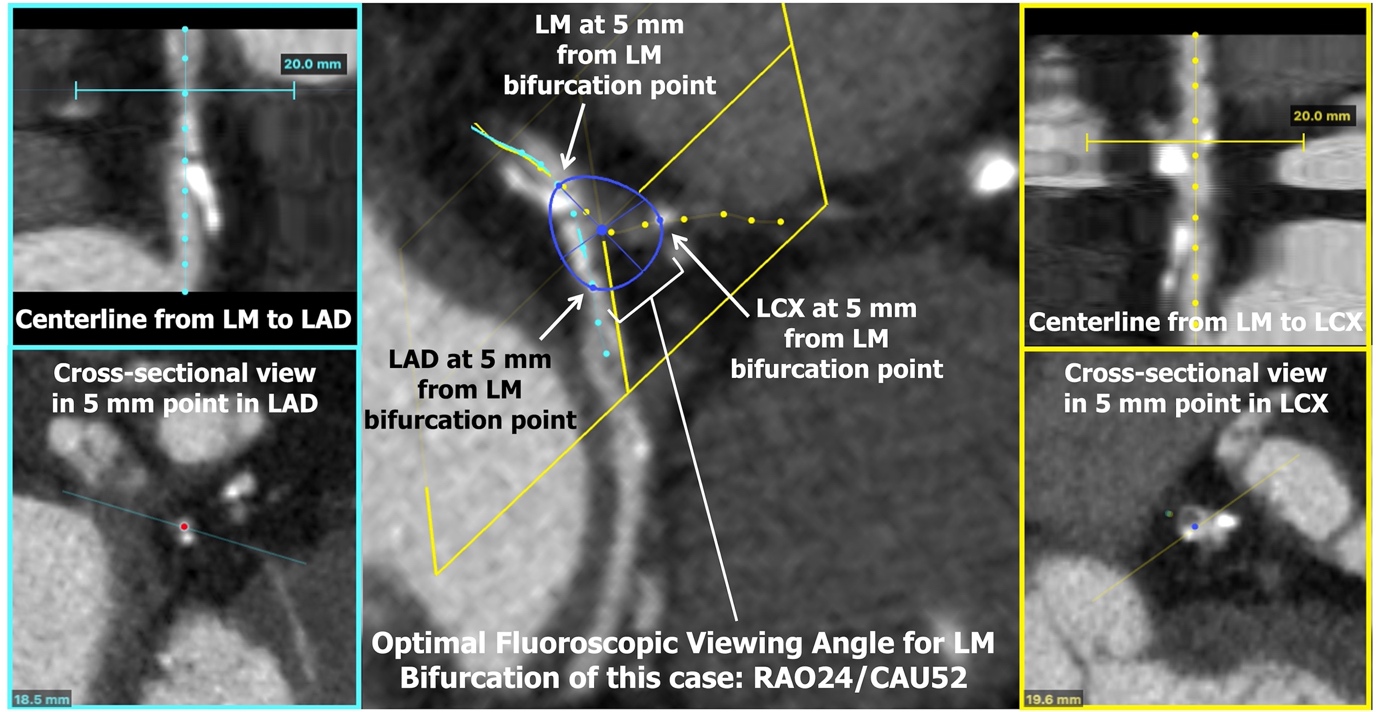
**

**Supplementary Fig. 4 Patient chart of µQFR analysis**

3VD = three-vessel disease; Other abbreviations as in **Supplementary Fig. 2**.

**
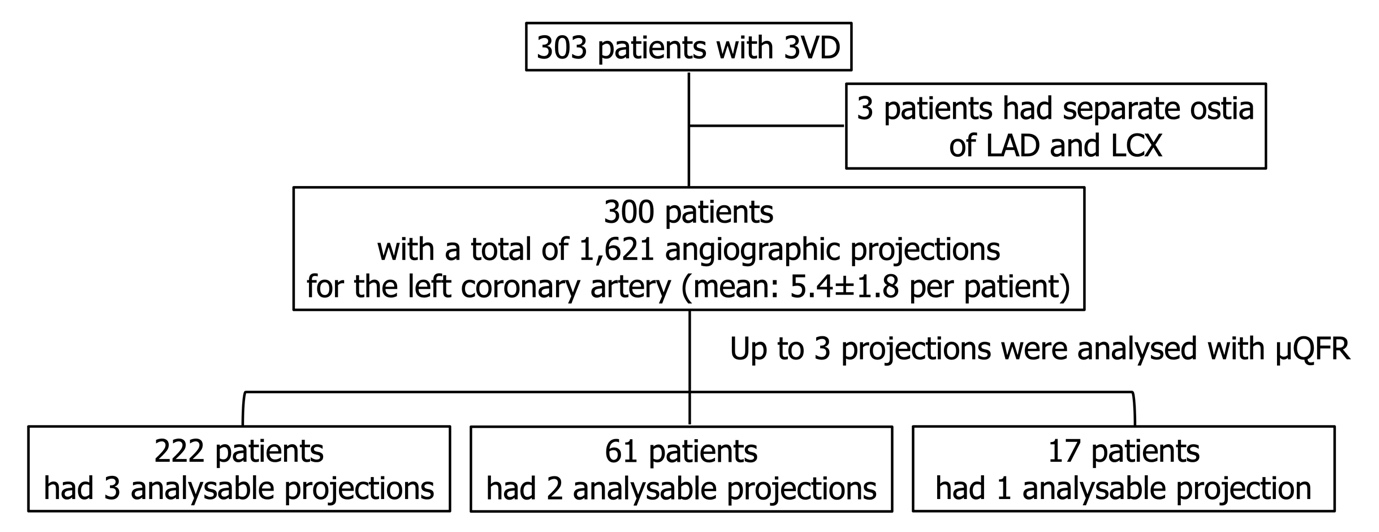
**

**Supplementary Fig. 5 The distribution of µQFR and FFR_CT_ in each anatomical landmark point**

**
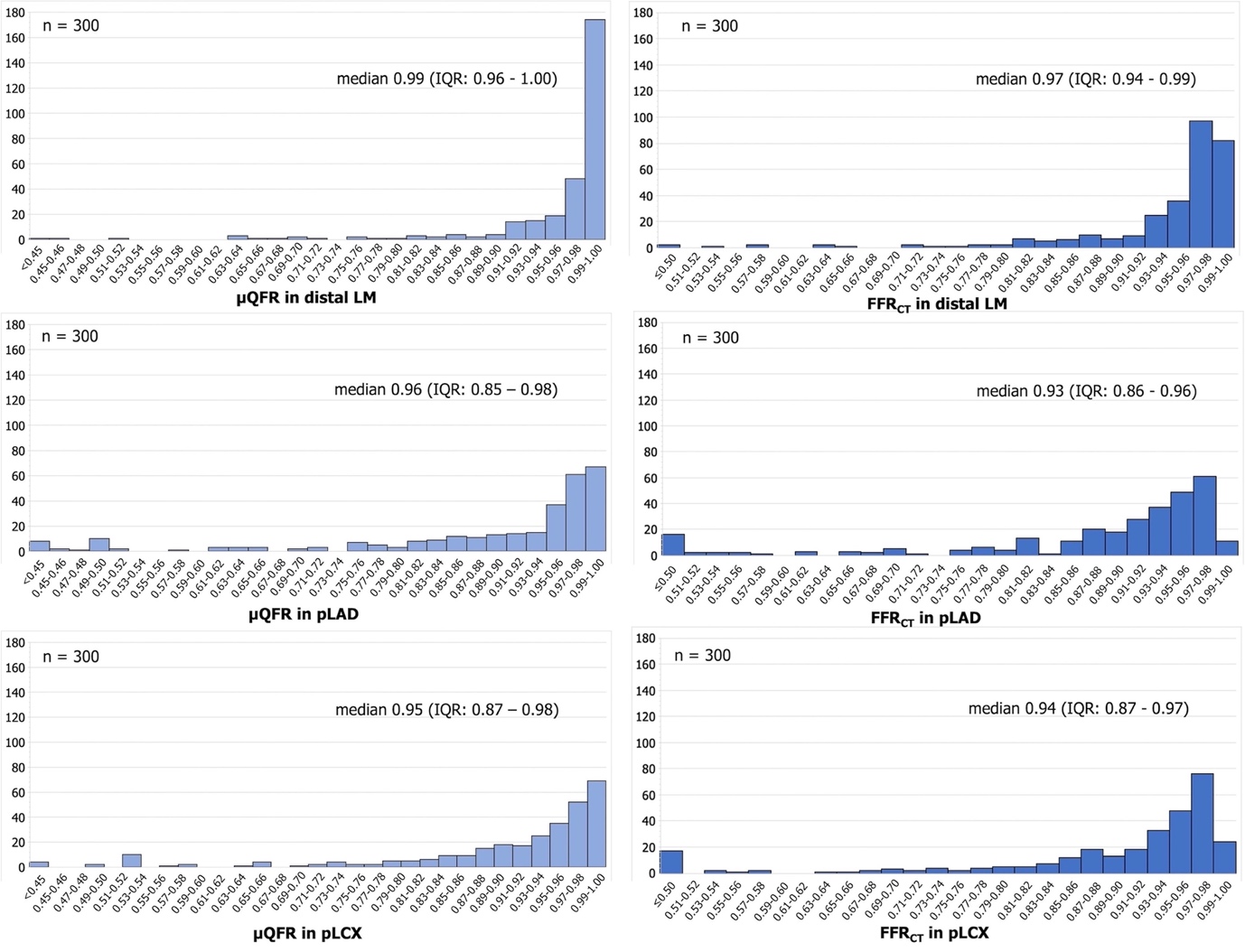
**

**Supplementary Fig. 6 Agreement between FFR_CT_ and µQFR on LM bifurcation analysis on the best and 2^nd^ fluoroscopic view: Bland-Altman plot of log-transformed data**

**
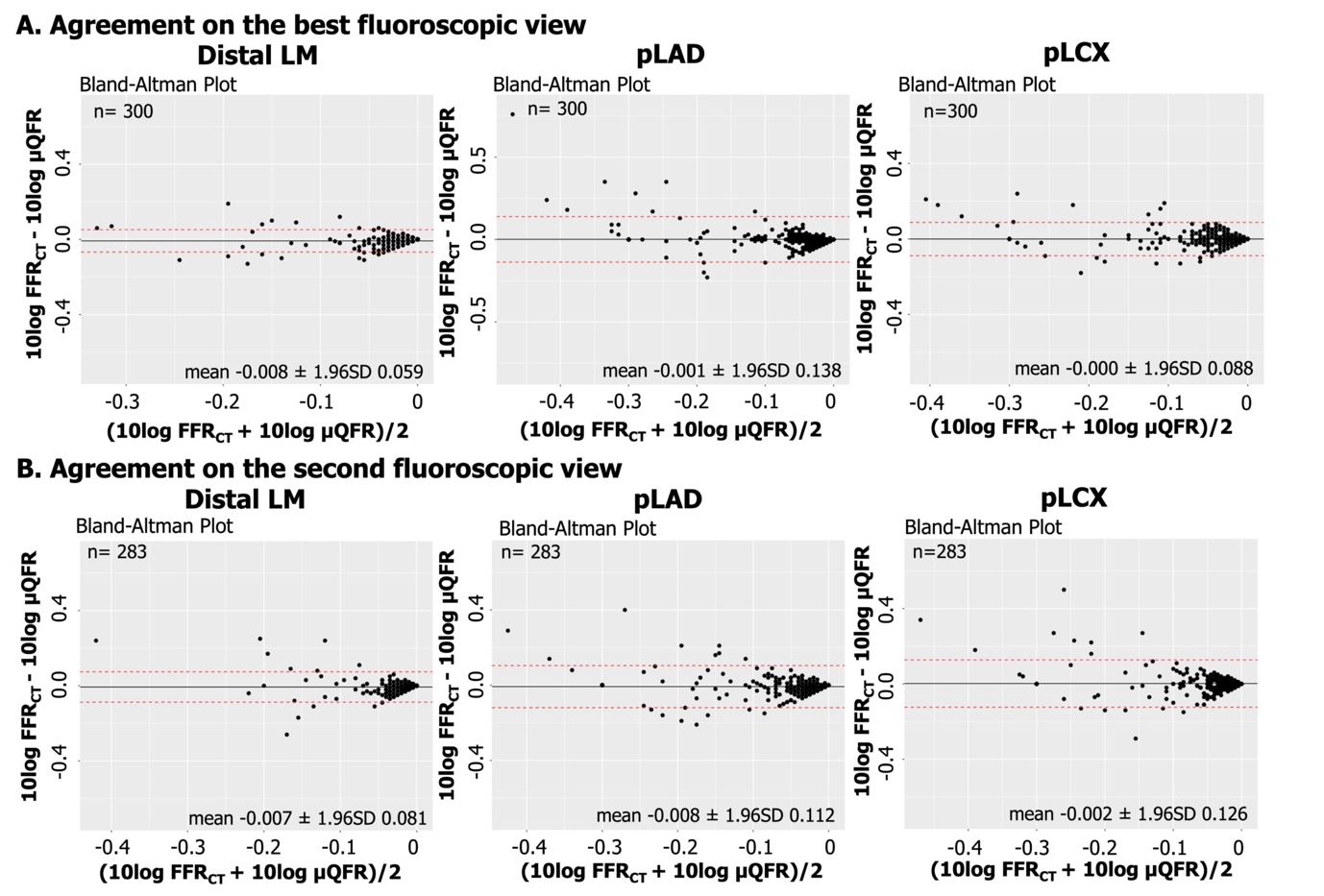
Supplementary Fig. 7 Reproducibility of µQFR analysis**


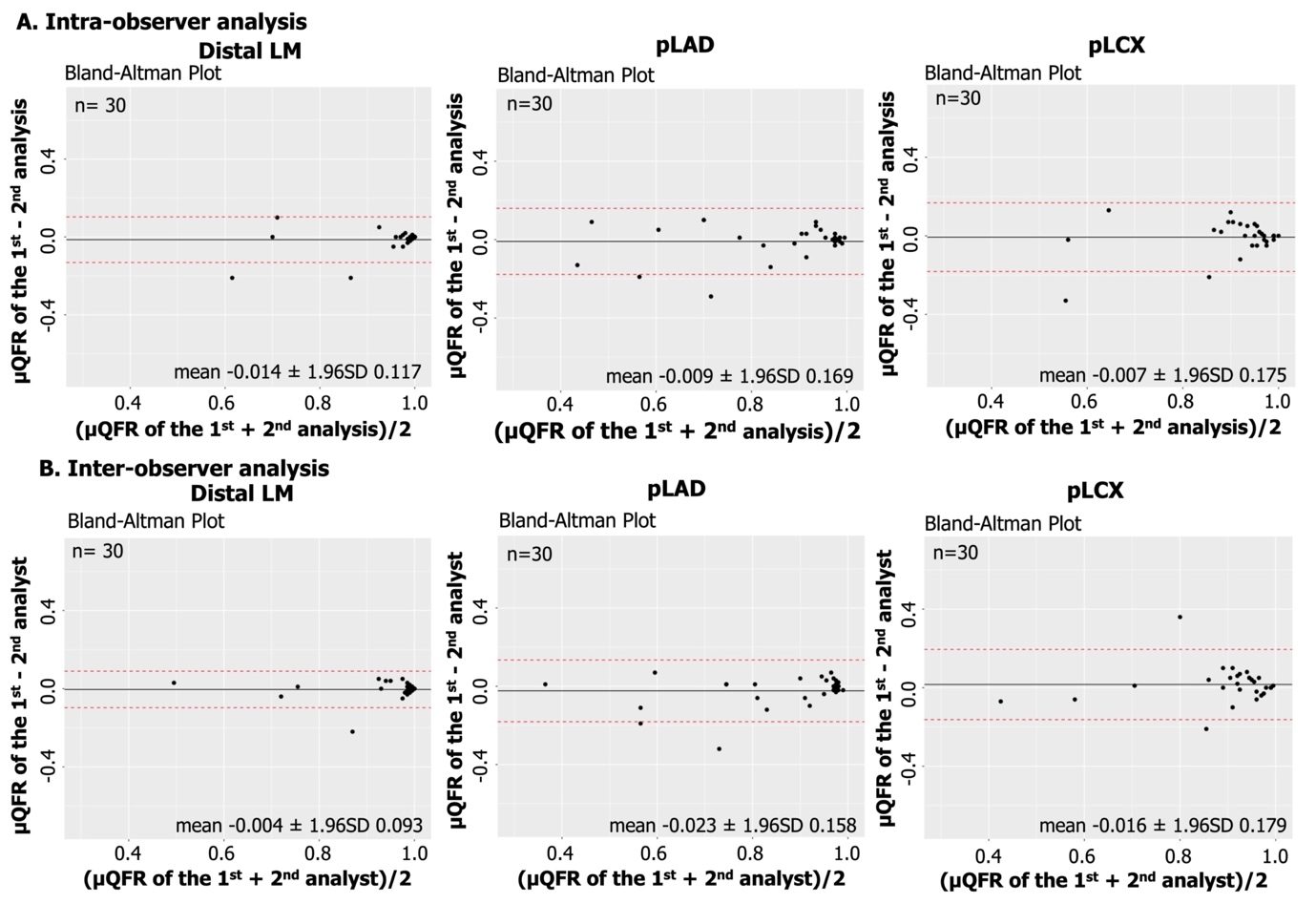

Supplement: Supplementary file 1 — Supplementary file1 (DOCX 1833 KB) [file 10554_2023_2974_MOESM1_ESM.docx]
